# *FLOWERING LOCUS T2* regulates spike development and fertility in temperate cereals

**DOI:** 10.1093/jxb/ery350

**Published:** 2018-10-05

**Authors:** Lindsay M Shaw, Bo Lyu, Rebecca Turner, Chengxia Li, Fengjuan Chen, Xiuli Han, Daolin Fu, Jorge Dubcovsky

**Affiliations:** 1 Department of Plant Sciences, University of California Davis, Davis, CA, USA; 2 State Key Laboratory of Crop Biology, Shandong Agricultural University, Tai’an, Shandong, China; 3 Department of Plant Sciences, University of Idaho, Moscow, ID, USA; 4 Howard Hughes Medical Institute, Chevy Chase, MD, USA

**Keywords:** Barley, *Brachypodium distachyon*, cereals, flowering, FLOWERING LOCUS, *T1 (FT1*), *FT2*, spike development, wheat

## Abstract

*FLOWERING LOCUS T2* (*FT2*) is the closest paralog of the *FT1* flowering gene in the temperate grasses. Here we show that overexpression of *FT2* in *Brachypodium distachyon* and barley results in precocious flowering and reduced spikelet number, while down-regulation by RNA interference results in delayed flowering and a reduced percentage of filled florets. Similarly, truncation mutations of *FT2* homeologs in tetraploid wheat delayed flowering (2–4 d) and reduced fertility. The wheat *ft2* mutants also showed a significant increase in the number of spikelets per spike, with a longer spike development period potentially contributing to the delayed heading time. In the wheat leaves, *FT2* was expressed later than *FT1*, suggesting a relatively smaller role for *FT2* in the initiation of the reproductive phase. *FT2* transcripts were detected in the shoot apical meristem and increased during early spike development. Transversal sections of the developing spike showed the highest *FT2* transcript levels in the distal part, where new spikelets are formed. Our results suggest that, in wheat, *FT2* plays an important role in spike development and fertility and a limited role in the timing of the transition between the vegetative and reproductive shoot apical meristem.

## Teaser

*FLOWERING LOCUS T2* (*FT2*) is expressed in the distal part of the developing wheat spike and contributes to the regulation of the number of spikelets per spike.

## Introduction

The time of year at which a plant flowers has an enormous influence on its reproductive success. For cereal crops, flowering outside the optimum time can significantly compromise grain yield and quality. The main seasonal signals used by temperate cereals to adjust flowering to an optimal reproductive time are the length of the night (photoperiod) and the exposure to extended periods of low temperatures (vernalization) (Distelfeld *et al.*, 2009). Wheat is a long day plant, and flowers earlier when the duration of the night is short or is interrupted by a short pulse of light (night break, Pearce *et al.*, 2017).

The pathways perceiving different environmental signals converge on the regulation of *FLOWERING LOCUS T* (*FT*) (Turck *et al.*, 2008). FT has been identified as a widely conserved regulator of floral induction, termed florigen, which is produced in the leaves and is transported to the shoot apical meristem (SAM) (Corbesier *et al.*, 2007; Jaeger and Wigge, 2007; Tamaki *et al.*, 2007). At its destination, FT forms a flowering activation complex by interacting with 14-3-3 proteins and the bZIP transcription factor FD (Pnueli *et al.*, 2001; Abe *et al.*, 2005; Wigge *et al.*, 2005; Taoka *et al.*, 2011; Li *et al.*, 2015). The 14-3-3 proteins are expressed in most tissues, where they act as a scaffold that links FT and FD. The more restricted expression of *FD* provides temporal and spatial specificity (Li *et al.*, 2015). This complex then interacts with promoters of a number of MADS box genes including *APETALA1* (*VRN1* in wheat), *FRUITFULL* (*FUL*) and *SUPPRESSOR OF OVEREXPRESSION OF CONSTANS 1* (*SOC1*), to initiate flowering (Abe *et al.*, 2005; Michaels *et al.*, 2005; Wigge *et al.*, 2005; Corbesier *et al.*, 2007; Mathieu *et al.*, 2007).

FT belongs to a family of small proteins characterized by a specific phosphatidylethanolamine (PE) binding protein (PEBP) domain. *In vitro*, FT binds to the diurnally changing phospholipid phosphatidylcholine (PC) which affects its ability to regulate flowering (Nakamura *et al.*, 2014). The PEBP gene family includes both activators and repressors of flowering (Pin *et al.*, 2010), which have been grouped into three main subfamilies: *TERMINAL FLOWER1* (*TFL1*)-like, *MOTHER OF FT* (*MFT*)-like and *FLOWERING LOCUS T* (*FT*)-like (Higgins *et al.*, 2010), which are conserved in cereals (Chardon and Damerval, 2005). *FT* belongs to the *FT*-like family, which includes at least six *FT*-like genes (*FT1* to *FT6*) in wheat (Faure *et al.*, 2007; Lv *et al.*, 2014; Halliwell *et al.*, 2016).

High transcript levels of *FT1* in temperate cereals are associated with accelerated flowering, suggesting a role in the promotion of flowering similar to Arabidopsis *FT* and rice *Hd3a* (Yan *et al.*, 2006; Lv *et al.*, 2014; Nitcher *et al.*, 2014; Ream *et al.*, 2014). Down-regulation of *FT1* by RNA interference (RNAi) resulted in non-flowering plants in *Brachypodium distachyon* and a 2–4 weeks flowering delay in hexaploid wheat mutants (Lv *et al.*, 2014). A similar delay of 25 d was observed in tetraploid wheat mutant plants combining a truncation mutation in *ft-A1* with an amino acid substitution in the *ft-B1* PEBP motif (P77S) (Lv *et al.*, 2014). This double mutant will be designated hereafter as *ft1*-mut rather than *ft1*-null because we currently do not know if it is a complete loss-of-function mutation. If *FT1* is not essential for wheat flowering, other *FT*-like genes may be involved in the induction of flowering in wheat (Shaw *et al.*, 2013; Lv *et al.*, 2014).

In wheat, *FT2* is the gene most similar to *FT1* (78% protein identity) suggesting a relatively recent duplication. Phylogenetic analyses suggest that this duplication occurred after the divergence between the grasses and Arabidopsis lineages, and that the *FT1–FT2* duplication in the grasses is independent of the *FT–TWIN SISTER of FT* (*TSF*) duplication in Arabidopsis (Yan *et al.*, 2006; Faure *et al.*, 2007; Li and Dubcovsky, 2008). Therefore, the study of the sub-functionalization of these genes requires independent experiments in the two lineages.

The *FT1* and *FT2* genes have received different names in different grass species. The closest homologs of wheat *FT1* and *FT2* in *B. distachyon* were originally designated as *FTL2* (Bradi1g48830) and *FTL1* (Bradi2g07070), respectively (Higgins *et al.*, 2010). However, *FTL1* was later re-designated as *BdFT2* and *FTL2* as *BdFT1* to match the phylogenetic relationships among the temperate grasses (Qin *et al.*, 2017). In this study, we will follow the later nomenclature. In rice, the closest homologs of wheat *FT1* are *Hd3* (*OsFTL2*) and *RFT1* (*OsFTL3*) and the closest to wheat *FT2* is *OsFTL1* (Izawa *et al.*, 2002).

In a QTL study in barley, polymorphisms in the *FT2* 3′-untranslated region were associated with small differences in heading time (1.2 d) (Wang *et al.*, 2010). In addition, this gene was detected in the SAM and its expression profile correlated with the up-regulation of *FT1* in the leaves and with multiple genes involved in inflorescence and floral organ development in the SAM (Digel *et al.*, 2015). Overexpression of *BdFT2* in *B. distachyon* (Wu *et al.*, 2013) or *OsFTL1* in rice (Izawa *et al.*, 2002) induced rapid flowering in transgenic calluses. In addition, overexpression of *OsFTL1* in rice resulted in reduced panicles or a single terminal flower (Izawa *et al.*, 2002). In this paper, we show that in the temperate cereals *FT2* plays an important role in spike development and fertility and a more limited role in the regulation of the initial transition of the SAM to the reproductive phase.

## Materials and methods

### Plant materials and phenotyping

The materials used in this study included the diploid *B. distachyon* Bd21-3, the barley variety Golden Promise (*Hordeum vulgare L.*), and the tetraploid wheat variety Kronos (*Triticum turgidum* ssp. *durum*). Bd21-3 has a weak vernalization requirement (2–3 weeks) and is photoperiod sensitive (Ream *et al.*, 2014). Golden Promise carries spring alleles at both the *Vrn-H1* and *vrn-H2* loci and the photoperiod-insensitive allele *ppd-H1*. Kronos carries the *Vrn-A1* allele for spring growth habit, a functional *vrn-B2* allele for winter growth habit, and the *Ppd-A1a* allele that confers earlier flowering under short days resulting in a reduced photoperiod response.

*Brachypodium distachyon* and barley plants were grown in a greenhouse at 25 °C and a long day (LD) photoperiod (16 h light–8 h dark) with a light intensity of 203 µmol m^−2^ s^−1^. *Brachypodium distachyon* seeds were vernalized at 4° C without light for 32 d. Wheat seeds were stratified at 4 °C for 2 d in the dark before germination. Mutant lines were evaluated in growth chambers set to 22 °C during daylight and 17 °C during dark periods. Lights were set to 260 µmol m^−2^ s^−1^ and were on for 16 h in LD experiments and 8 h in short day (SD) experiments.

Mutant and wild-type lines of tetraploid wheat variety Kronos were compared in two field trials. For the 2015 field trial, a minimum of 25 plants per line were sown on 7 January 2015 and heading time was recorded for each individual plant as the number of days from planting to full emergence of the main spike. For the 2017 field trial, seeds were sown on 18 November 2016 in a randomized block design (eight blocks) and heading time was recorded when 50% of the plants showed fully emerged spikes. For the 2017 trial, spike traits were measured from four plants per row for a total of eight rows per genotype (except for the wild-type, from which we measured four rows due to poor germination in the other four rows). Average spike length (SPL) was measured from the base of the spike to the tip of the terminal spikelet, excluding awns. The number of spikelets per spike (SPS) was determined by adding the number of fertile and non-fertile spikelets. The number of florets per spikelet (FLS) was determined from the two middle spikelets of the spike. Spikes were threshed, grain number was counted, and the average grain weight was calculated by dividing the total grain weight by the number of grains per spike (GRS). Seed setting rate (fertility) was calculated as the number of grain per spike divided by the number of florets per spike.

### Plasmid construction and transformation

Constructs and protocols to overexpress or down-regulate *FT2* are described in Supplementary Fig. S1 at *JXB* online. Tissue culture and shoot regeneration were conducted in a growth chamber under 23 °C, LD, and low light intensity (52 µmol m^−2^ s^−1^). Putative transgenic plants were confirmed by PCR and resistance to 0.3% (v/v) Finale® herbicide.

### Development of Kronos *ft2*-null mutants

From a sequenced EMS mutant population in the tetraploid wheat variety Kronos (Krasileva *et al.*, 2017), we selected a donor splice-site mutation in *FT-A2* (T4-684) and a premature stop codon in *FT-B2* (T4-2493). *FT*-A2 alleles were genotyped by digesting a PCR fragment amplified with primers FT2AF3 (5′-GAATCTCCTGCACAAACTTAATCATCAA-3′) and FT2AR2 (5′-CCTGTATAATCGACTTCATCCTCACC-3′) with restriction enzyme *Rsa*I. *FT-B2* alleles were genotyped by digesting a PCR fragment amplified with primers FT2BF2 (5′-GGCTGCTTGACAACCATTGTGT-3′) and FT2BR2 (5′-CCTGTATAAACCACCACCCATACG-3′) with restriction enzyme *Bsr*I. Mutant plants were backcrossed twice to Kronos to reduce background mutations and then intercrossed to generate a double mutant (*ft-A2/ft-B2*, henceforth designated as *ft2*-null). These BC_2_F_1_ hybrids heterozygous for both mutations were self-pollinated to generate a BC_2_F_2_ segregating population, from which plants homozygous for all four *FT-A2* and *FT-B2* allele combinations were selected.

We then intercrossed the *ft2*-null mutant with *ft1*-mut to study the effect of *FT2* in plants with limited *FT1* function. After two backcrosses to Kronos, we selected all four possible homozygous combinations of the *FT-A2* and *FT-B2* alleles in the *ft1*-mut background. We also selected a line homozygous for wild-type *FT1* and *FT2* alleles to be used in control experiments to study *FT1* function in comparison with *ft1*-mut. The homozygous quadruple mutant (*ft1*-mut/*ft2*-null) was not recovered from the BC_2_F_2_ segregating population so we used a quadruple mutant recovered from an earlier segregating population.

### Gene expression analysis

In wheat, leaf tissue for gene expression analysis in the mutant lines was harvested in the morning after 3 h of light and immediately frozen in liquid nitrogen. The number of replications in each experiment are indicated in the legends of the respective figures. RNA was extracted using the Spectrum Plant Total RNA kit (Sigma-Aldrich) or TRIzol® Reagent (Life Technologies) following the manufacturer’s instructions. RNA was then treated with DNase I (Roche) and cDNA was synthesized using the High Capacity Reverse Transcription Kit (Applied Biosystems).

Primer sequences for *B. distachyon* and wheat *FT*-like genes were reported previously (Lv *et al.* 2014). Primers for barley are listed in Supplementary Table S1, primer sequences for *Ppd1* are from Shaw *et al.* (2012), and primer sequences for the control gene *ACTIN* are from Pearce *et al.* (2017). Quantitative RT-PCR was performed using VeriQuest Fast SYBR Green qPCR Master Mix in a 7500 Fast Real-Time PCR system (Applied Biosystems). *ACTIN* was included as the endogenous control gene for all reactions and transcript levels were expressed as fold-*ACTIN* using 2^–Δ*C*T^.

### Statistical analyses

Averages were compared using one-way ANOVAs for single locus and two-way factorial ANOVAs for two or more loci. Homogeneity of variances was tested using the Levene’s test and normality of residuals with the Shapiro–Wilks test. Data was transformed when necessary to meet the ANOVA assumptions. When transformations were not found to restore normality, non-parametric tests were used (e.g. Kruskal–Wallis). All statistical analyses were performed using SAS 9.4 (SAS Institute).

## Results

### Overexpression of FT2 promoted rapid flowering in *B. distachyon* and barley

*Brachypodium distachyon* (*Bd*) transgenic calli overexpressing *BdFT2* under the maize *UBIQUITIN* promoter (Ubi::BdFT2; Supplementary Fig. S1A) or the 35S promoter (35S::BdFT2:GFP; Supplementary Fig. S1B) were grown under LD conditions. The T_0_ transgenic plants regenerated shoots and developed floral organs within 1 week of being transferred to the regeneration medium (Supplementary Fig. S2A, B), whereas the non-transgenic regenerated plants showed normal flowering (Supplementary Fig. S2C). The extremely rapid flowering under LD conditions resulted in plants with few leaves, very short spikes with one or few spikelets and no seeds. Transgenic plants kept under SD conditions for 1 week before being transferred to LD conditions had a few more leaves than in the LD experiment, but they also flowered within 1 week of being transferred to LD conditions and failed to set seeds.

Overexpression of the wheat *FT-D2* homolog under the maize *UBIQUITIN* promoter (Ubi::FT-D2:Myc Supplementary Fig. S1C) in the barley variety Golden Promise resulted in very early flowering and produced a single tiller with only six leaves. The spikes from these plants were short, with one or few spikelets (Supplementary Fig. S2D, E). Since the *B. distachyon* and barley T_0_ transgenic plants failed to yield any seeds, we were not able to do any additional analyses.

### Down-regulation of FT2 delayed heading time and reduced grain production in B. distachyon and barley

**Fig. 1.**
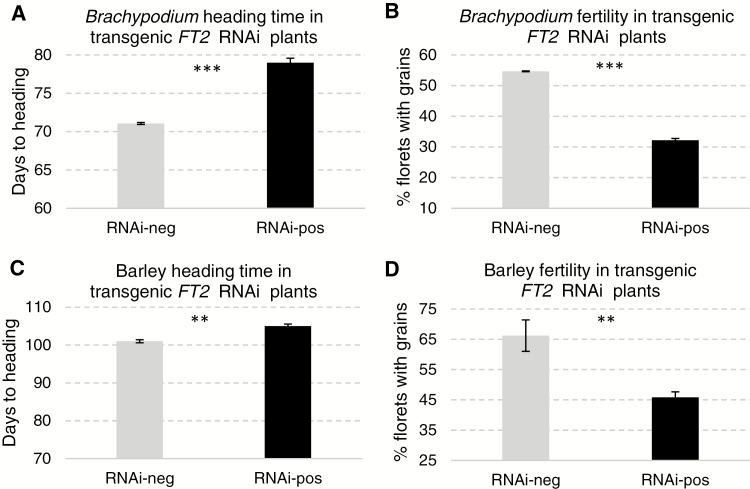
*Brachypodium distachyon* and barley RNAi transgenic plants. (A) Average heading time of *B. distachyon FT2*_*RNAi*_-positive plants (*n*=9) and *FT2*_*RNAi*_-negative control plants (*n*=18). (B) Average percentage of florets with grains (same genotypes and number of replications as in (A)). (C) Average heading time of barley *FT2*_*RNAi*_-positive plants (*n*=61) and *FT2*_*RNAi*_-negative control plants (*n*=4). (D) Average percentage of florets with grains (same genotypes and number of replications as in (C)). Error bars indicate standard errors of the means. **P<0.001, and ***P<0.0001.

To study the effect of the reduced *FT2* expression, we developed RNAi constructs containing *B. distachyon* and barley *FT2*-specific fragments (Ubi::FT2RNAi, Supplementary Fig. S1D). The selected fragments did not share more than 18 identical consecutive nucleotides with other *FT*-like genes to avoid co-silencing. In *B. distachyon*, we recovered seeds from three positive T_0_ transgenic *B. distachyon* plants that were designated as RNAi-678, -693 and -700. The T_1_ progeny of these plants showed reductions in *FT2* transcript levels that varied between 83.6% (RNAi-678) and 91.8% (RNAi-700) relative to the non-transgenic sibling plants (Supplementary Fig. S3A). Among the 27 T_1_ seeds recovered from the T_0_ plants, we found only nine plants carrying the transgene. This number is significantly lower than the expected 20 plants carrying the transgene based on a 3:1 segregation ratio (χ^2^=25.0, *P<*0.0001). This segregation distortion suggest that the transgene could be affecting gamete or seedling viability. On average, the *FT2*_*RNAi*_ transgenic *B. distachyon* plants headed 8 d later (*P=*0.004) and showed a 25.9% reduction in fertility (*P<*0.017) relative to their non-transgenic sibling control (NTSC) (Fig. 1A, B; Supplementary Table S2).

In barley, we recovered 11 transgenic plants from the transformation with the *HvFT2*_*RNAi*_ construct (Supplementary Fig. S1D). In the transgenic T_1_ plants, the *FT2* transcript levels were reduced on average by 95.1% relative to their NTSC (Supplementary Fig. S3B). On average, the *FT2*_*RNAi*_ transgenic barley plants headed 4 d later than the NTSC (*P=*0.0023, Fig. 1C; Supplementary Table S3). No significant differences were detected in the number of florets per spike (*P=*0.6272) but a significant reduction was observed in the number of grains per spike, resulting in a 20.6% reduction in fertility (*P=*0.0003) relative to the NTSC (Fig. 1D; Supplementary Table S3).

### Down-regulation of *FT2* by RNAi was associated with the down-regulation of other *FT*-like genes and *VRN1* in *B. distachyon* and barley

In both *B. distachyon* and barley, the down-regulation of *FT2* by RNAi was associated with the down-regulation of other members of the *FT*-like family and *VRN1*, a downstream target of *FT1*. In *B. distachyon*, the Ubi::BdFT2_RNAi_ plants showed reductions in transcript levels relative to the non-transgenic sibling lines for all the *FT*-like genes. These differences were significant for *FT2* (88%, *P<*0.0001), *FT4* (51%, *P=*0.0324), *FT5* (60%, *P=*0.0003), and *VRN1* (56%, *P=*0.0122), but not for *FT1* (38%, *P=*0.0760) or *FT3* (40%, *P=*0.2788) (Supplementary Fig. S4A).

In barley, the Ubi::HvFT2_RNAi_ plants showed significant reductions in the transcript levels of *FT1* (86%, *P<*0.0001), *FT2* (95%, *P<*0.0001), *FT3* (71%, *P=*0.0003), and *VRN1* (35%, *P=*0.0105) relative to the non-transgenic sibling lines. No significant differences were detected for *FT4* and *FT5* (Supplementary Fig. S4B).

### A mutation in *ft-A2* in tetraploid wheat was associated with small delays in heading time

**Fig. 2.**
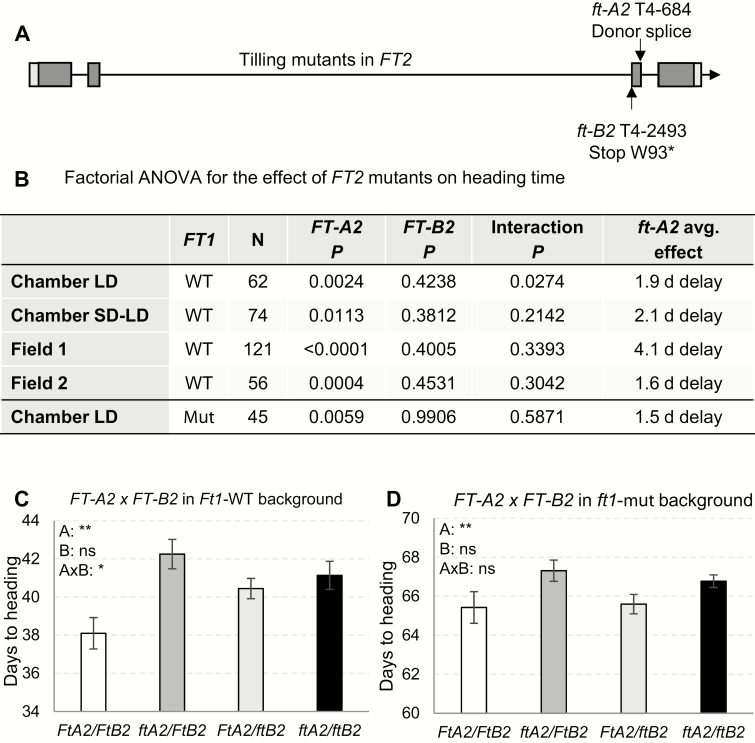
Effect of *FT2* mutants on heading time. (A) Mutations selected in a Kronos mutant population (Krasileva *et al.*, 2017) for *FT-A2* (TraesCS3A01G143100) and *FT-B2* (TraesCS3B01G162000). The PEBP domain, which extends along most of the *FT2* gene, is represented by dark grey rectangles. (B) Two-way factorial ANOVAs for the four homozygous combinations of mutant and wild-type alleles at the *FT-A2* and *FT-B2* loci. Three experiments were done in growth chambers and two in the field. Two chamber experiments were performed under long days (LD), the first with plants carrying the *FT1* wild-type (WT) allele and the second in the *ft1*-mut background. In the other growth chamber experiment, plants were grown for 7 weeks under SD and then moved to LD (SD–LD). (C, D) Average heading time of plants segregating for *FT-A2* and *FT-B2* in growth chambers under LD. The *P* values for the different mean comparisons in (C) and (D) are in the first and last rows of the table in (B), respectively. (C) Segregation of *FT2* in an *Ft1* wild-type allele background (*n*=10–22 plants per genotype). (D) Segregation of *FT2* in an *ft1*-mut mutant allele background (*n*=7–19 plants per genotype). Error bars indicate standard errors of the means. A: *FT-A2* effect; B: *FT-B2* effect; A×B: interaction *FT-A2*×*FT-B2.*

For *FT-A2*, we selected mutant line T4-684 (henceforth *ft-A2*), which carries a mutation in the donor splice site of the third intron (Fig. 2A). We sequenced *FT-A2* transcripts from the leaves of mutant line T4-684 and confirmed that the modified splicing site resulted in transcripts that encode five missense amino acid changes followed by a premature stop codon that eliminates the distal 74 amino acids (41.6%). For *FT-B2*, we selected mutant line T4-2493 (henceforth *ft-B2*), which includes a premature stop codon (W93*) that is expected to eliminate the distal 48.3% of the protein (Fig. 2A). The predicted truncations in these two proteins include conserved portions of the PEBP domain (Ahn *et al.*, 2006), and are not expected to be functional.

We selected at least 10 BC_2_F_4_ plants for each of the four possible homozygous combinations of the *FT-A2* and *FT-B2* alleles from the population segregating for both genes (Fig. 2B) and tested them in two growth chamber experiments and two field trials. One chamber experiment was conducted under LD and the other one under SD conditions for 7 weeks followed by LD conditions. In all four experiments, the *ft-A2* mutation was associated with small but significant delays in heading time (2–4 d) and the *ft-B2* mutation showed no significant effects (Fig. 2B). The interactions between the two *FT2* homeologs were not significant in three experiments and marginally significant (*P=*0.03) in the LD chamber experiment (Fig. 2B, C).

We also tested if the *ft2*-null mutants affected the total numbers of leaves in a greenhouse experiment under LD conditions. We found no significant differences (*P=*0.44) in the total number of leaves produced by plants carrying the *Ft2* wild-type alleles (23.2 ± 1.1, *n*=15) and those carrying the *ft2*-null alleles (24.5 ± 1.2, *n*=14). Since no more leaves are produced once the SAM transitions to the reproductive stage, this result suggests a limited role of *FT2* in the regulation of the transition between the vegetative and reproductive SAM.

To test if the effect of *FT2* on heading time was dependent on or masked by the presence of *FT1*, we evaluated a separate set of lines carrying the same four homozygous *FT2* allele combinations in an *ft1*-mut background in a growth chamber under LD conditions. In the *ft1*-mut background, the *ft-A2* mutation was associated with a 1.4–1.9 d delay in heading time (*P=*0.0059), whereas the *ft-B2* allele showed no significant effect (Fig. 2B, D). These results were similar to the delay in flowering caused by the *ft2* mutants in plants carrying the wild-type *Ft1* allele.

As a control, we tested the effect of *FT1* on heading time in plants homozygous for the *Ft2* wild-type alleles. Plants homozygous for the *ft1*-mut alleles flowered on average 21 d later than plants homozygous for the *Ft1* wild-type alleles (*P* <0.0001, Supplementary Table S4). This difference was consistent with the 25–27 d difference in heading time observed between the respective genotypes in Fig. 2C (*Ft1*) and Fig. 2D (*ft1*), and with the 25 d difference reported in a previous study comparing the same *ft1*-mut and wild-type Kronos plants (Lv *et al.*, 2014).

In summary, our results showed that the effect of *FT2* mutation on Kronos heading time was roughly 10-fold smaller than the effect of *FT1* and that, among the *FT2* mutants, the effect of *ft-A2* on heading time was stronger than the effect of *ft-B2*.

### Effect of *ft2* mutations on spike development in tetraploid wheat

**Fig. 3.**
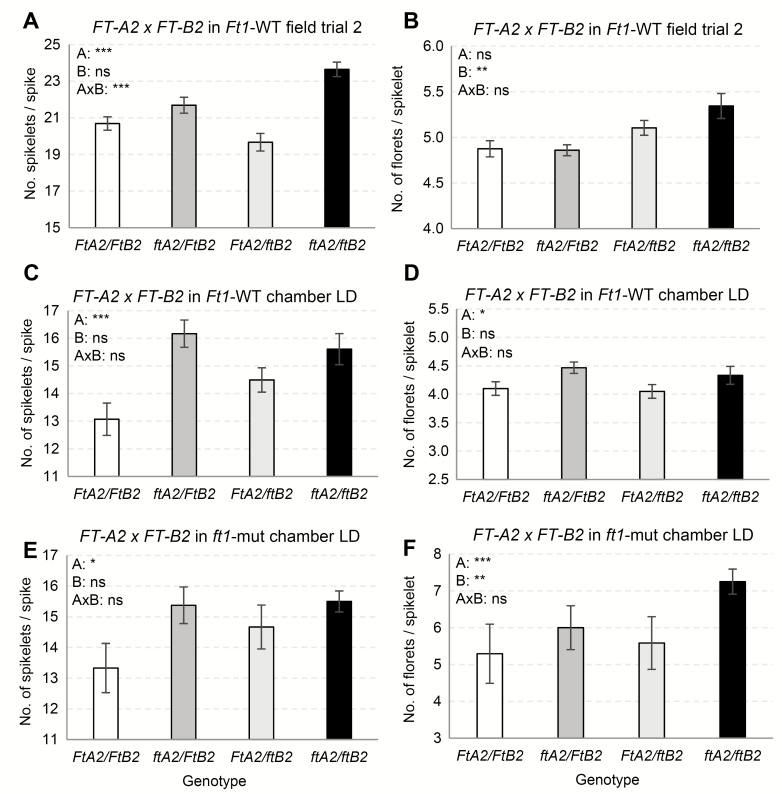
Effect of *FT2* mutants on spike development. (A, C, E) Number of spikelets per spike (SPS). (B, D, F) Number of florets per spikelet (FLS). (A, B) Field experiment (*n*= 4–8, *Ft1* wild-type allele). Measurements were taken from the main spike from four plants per row, with four to eight rows per genotype (Supplementary Table S7). (C, D) Growth chamber experiment under LD (*n*= 6–12, *Ft1* wild-type allele). Measurements were taken from the three largest spikes per plant (Supplementary Table S6). (E, F) Growth chamber experiment under LD (*n*= 6–8, *ft1*-mut allele). Measurements were taken from the main spike per plant. Error bars indicate standard errors (Supplementary Table S5). Significance of the statistical analyses from Supplementary Tables S5–S7 are summarized in each graph using the letters A for the *FT-A2* effect, B for the *FT-B2* effect, and A×B for the *FT-A2×FT-B2* interaction.

We also investigated the effect of the four homozygous *FT2* allelic classes on spike development in the different *FT1* backgrounds using factorial ANOVAs. In all three experiments, the *ft-A2* mutant allele was associated with significant increases in the number of spikelets per spike (2–3 SPS or 10–15% increase), and the *ft-B2* allele was associated with non-significant increases (2–5%, Table 1). The increases in spikelet number in the *ft2*-null mutant relative to the wild-type (14–19%) were larger than the increases for the single mutants, which suggests that the *FT-B2* gene has a residual effect on SPS (Table 1; Fig. 3; Supplementary Tables S5–S7).

**Table 1.** Effect of single and double *ft2* mutations on spikelet traits

				Effect (%)^*a*^		
	**Experiment**	***FT1***	*n*	***ft-A2***	***ft-B2***	***ft-A2*/*ft-B2***
Spikelets/spike	Chamber LD	Mut	26	+10.3*	+5.1	+16.3***
	Chamber LD	WT	37	+15.3***	+2.9	+19.4**
	Field	WT	28	+12.3***	+2.2	+14.3*
Florets/spikelet	Chamber LD	Mut	26	+21.8***	+13.7**	+37.0*
	Chamber LD	WT	37	+8.0*	−2.1	+5.7
	Field	WT	28	+2.2	+7.3**	+9.6***
Fertility	Chamber LD	Mut	26	−15.7	−47.8**	−72.9*
	Chamber LD	WT	37	−8.9	−14.6	−23.8
	Field	WT	28	−16.7***	−19.1***	−32.3
Grains/spike	Chamber LD	Mut	26	+0.1	−44.3*	−61.2
	Chamber LD	WT	37	+10.0	−32.5	−6.0
	Field	WT	28	−5.3	−11.7**	−15.5

^*a*^ Main effects from the factorial ANOVA. Complete ANOVAs in Supplementary Tables S5–S7. + and − indicate higher and lower values in the mutant relative to the wild-type allele, respectively ((mutant−WT)/mutant).

*P<0.05, **P<0.01, ***P<0.001: probability from *t*-test between homozygous wild-type and mutant classes.

We also observed significant increases in the number of florets per spikelet (FLS; 2.2–21.8%) in two out of the three experiments for both *FT-A2* and *FT-B2* (Table 1; Supplementary Fig. S5). In five out of the six possible comparisons, the percentage increases in FLS in the double *ft2*-null mutant were larger than in the single *FT-A2* or *FT-B2* mutants (Table 1; Fig. 3; Supplementary Tables S5–S7).

The *ft-A2*, *ft-B2* and *ft2*-null mutations were associated with consistent reductions in fertility across all conditions, however due to the large variability observed for this trait, the differences were significant only in the field experiment and in the chamber experiment in the absence of *FT1* (Table 1). The larger reductions in fertility associated with *ft-B2* were also reflected in a consistent reduction in the number of grains per spike (GRS; 12–44%, significant in two experiments). The *ft-A2* mutant had a non-significant effect on GRS, likely due to the opposite effects of sterility and increased SPS (Table 1; Supplementary Tables S5–S7). In the *ft2*-null mutants, the combined effect was a reduction of 6–61% in GRS relative to the wild-type. Taken together, these results suggest that *FT2* affects SPS, FLS, GRS, and fertility, and that, with the exception of SPS, these effects are larger in the absence of *FT1*.

As a control, we looked at the effect of the *FT1* mutants on the different spike traits. We observed that the spikes of the *ft1*-mut plants had a significant reduction in fertility (*P=*0.0003) and grains per spike (*P=*0.0319) and a significant increase in florets per spike (*P=*0.0228) relative to the spikes of the *Ft1* wild-type plants (Supplementary Table S4). These results indicate that *FT1* also plays a role in spike development and fertility.

### Expression of FT2 in the leaves of tetraploid wheat

Qin *et al.* (2017) reported the presence of two *FT2* alternative splice forms in the leaves of *B. distachyon*, barley and wheat, one encoding the complete FT2 protein and the other a shorter splice variant. In this study, we also observed a shorter splice variant in the leaves of tetraploid wheat of similar size to the one reported for *B. distachyon* for both *FT-A2* and *FT-B2* (Supplementary Fig. S6). However, the short variant was detected at lower levels than the splice variant encoding the complete gene both in 3- and 6-week-old plants (Supplementary Fig. S6). In addition, we detected mostly the longer splice variant in the vast majority of our RNA samples, suggesting that an *FT-A2* alternative splicing mechanism has limited biological relevance in wheat. Therefore, in the following qRT-PCR experiments we used primers that amplify both splice variants.

**Fig. 4.**
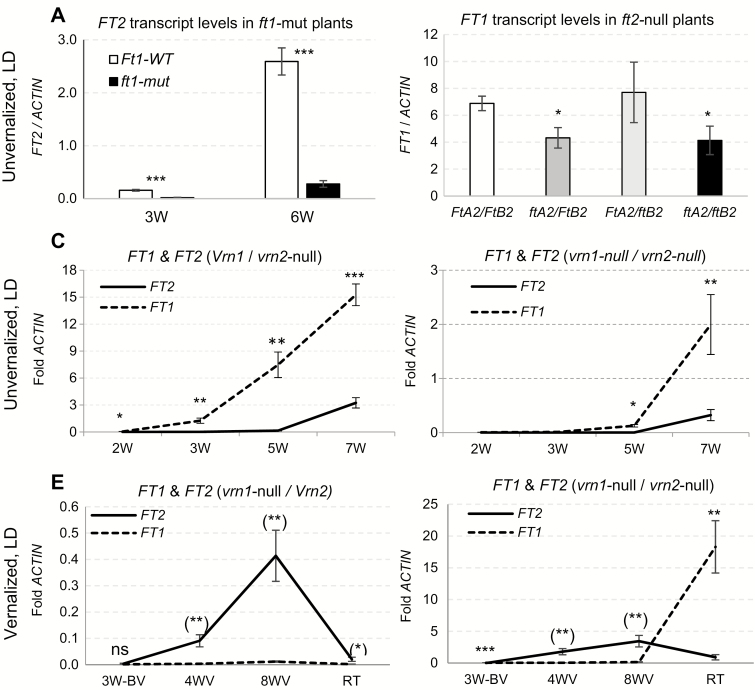
Relative expression of *FT1* and *FT2* in the leaves of tetraploid wheat Kronos under LD. (A–D) Unvernalized plants under LD. (A) *FT2* transcript levels in *ft1*-mut mutants in 3- and 6-week-old plants (3W and 6W). (B) *FT1* transcript levels in *ft2* mutants in 5-week-old plants. Asterisks indicate significance in a factorial ANOVA. *FT1* and *FT2* transcript levels in (C) Kronos *Vrn1*/*vrn2*-null mutant and (D) Kronos *vrn1-null*/*vrn2*-null mutant. (E, F) Plants vernalized at 4°C and grown under LD. BV, before vernalization; V, during vernalization; RT, 2 weeks after returning plants to room temperature. *FT1* and *FT2* transcript levels in (E) Kronos *vrn1*-null (*vrn1*-null/*Vrn2*) and (F) Kronos *vrn1*-null/*vrn2*-null. Error bars indicate standard errors. *P<0.05, **P<0.01, ***P<0.001; ns, not significant. Parentheses around the asterisks indicate that *FT2*>*FT1.* The absence of parentheses indicates that *FT1*>*FT2*.

In 3- and 6-week-old Kronos lines grown under LD conditions, we observed a significant decrease in *FT2* transcript levels in the leaves of the *ft1*-mut mutants relative to the plants carrying the wild-type *Ft1* allele (Fig. 4A). A reciprocal, but much smaller reduction in *FT1* transcript levels was associated with the *ft-A2* mutant allele in a factorial ANOVA including the four *FT2* allelic classes (*P=*0.0401; Fig. 4B). In a separate qRT-PCR experiment, we also observed down-regulation of *FT1* and *VRN1* transcripts in the *ft2*-null mutant relative to the wild-type, but the differences were significant only for *VRN1*. No significant differences in transcript levels were detected for *FT3*, *FT4*, or *FT5* between the same genotypes (Supplementary Fig. S4C).

To understand the roles of *FT1* and *FT2* and their regulation by other flowering genes, we compared their expression profiles during development in different environments and genetic backgrounds. In Kronos *vrn2*-null plants grown under LD conditions without vernalization, *FT2* was up-regulated 2–3 weeks after the up-regulation of *FT1*, in the presence of both wild-type (Fig. 4C) and mutant *VRN1* alleles (Fig. 4D; Chen and Dubcovsky, 2012). The earlier expression of *FT1* in the leaves relative to *FT2* is consistent with the stronger role of *FT1* in the initiation of the reproductive phase. In this experiment the *FT1* and *FT2* transcript levels were at least 8-fold higher in plants carrying the functional *Vrn1* allele (Fig. 4C) than in those carrying *vrn1*-null mutant alleles (Fig. 4D). Since these plants were homozygous for the *vrn2*-null allele, this result suggests that *VRN1* can act as a promoter of *FT1* and *FT2* transcription in the leaves independently of *VRN2*.

We then studied the expression of *FT1* and *FT2* in Kronos *vrn1*-null plants before, during, and after vernalization in the presence (Fig. 4E) and absence (Fig. 4F) of the *VRN2* repressor (Chen and Dubcovsky, 2012). In both genetic backgrounds, *FT1* transcripts were not detected during vernalization whereas *FT2* showed a significant up-regulation during vernalization and a down-regulation when plants were returned to room temperature (Fig. 4E, F). We have shown before that *VRN2* transcripts increase rapidly when *vrn1*-null plants are returned to room temperature (Chen and Dubcovsky, 2012). This may explain why at this time point the transcript levels of *FT1* remained low in the plants carrying a functional *Vrn2* allele (Fig. 4E) and increased rapidly in the *vrn2*-null plants (Fig. 4F). Similarly, transcript levels of *FT2* during vernalization were approximately 10-fold lower in the presence of a functional *Vrn2* allele (Fig. 4E) than in its absence (Fig. 4F). Taken together, these results indicate that VRN2 acts as a transcriptional repressor of both *FT1* and *FT2*, that *FT2* can be up-regulated during vernalization independently of *VRN1* and *VRN2*, and that the up-regulation of *FT1* at room temperature does not result in a rapid up-regulation of *FT2* (Fig. 4F).

**Fig. 5.**
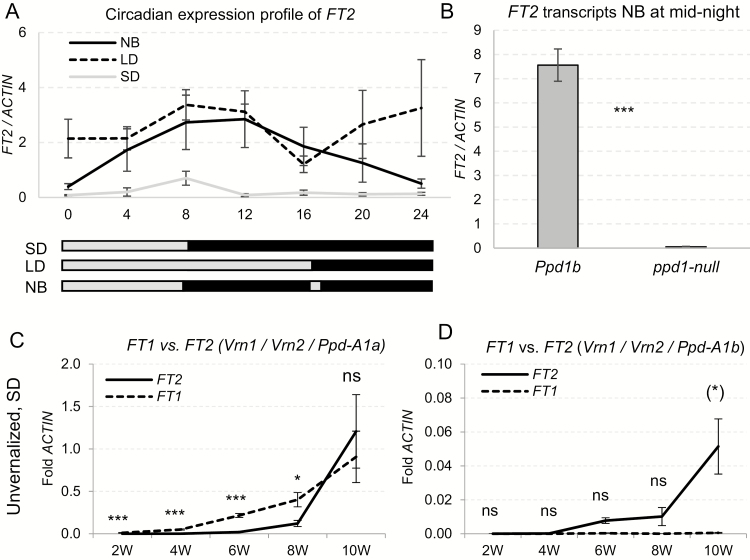
*FT1* and *FT2* transcript levels in different genetic backgrounds and environments. (A) Circadian expression profile of *FT2* under continuous light in Kronos-*Ppd1b* plants grown for 4 weeks under SD, and transferred for 3 weeks to LD, night break (NB) or SD (*n*=6). (B) *FT2* expression in the middle of the night before the NB light pulse in Kronos-*Ppd1b* and Kronos-*ppd1*-null mutants grown for 4 weeks in SD, followed by 6 weeks under NB conditions (*n*=6). (C, D) *FT1* and *FT2* transcript levels in unvernalized plants grown under SD in (C) Kronos-*Ppd1a* and (D) Kronos-*Ppd1b*. Error bars are standard errors. *P<0.05, **P<0.01, ***P<0.001; ns, not significant. Parentheses around the asterisks indicate that *FT2*>*FT1.* The absence of parentheses indicates that *FT1*>*FT2*.

Finally, we explored the effect of photoperiod on the transcript levels of *FT1* and *FT2* in Kronos lines carrying different *PPD1* alleles (Fig. 5). We grew Kronos plants carrying the photoperiod-sensitive *Ppd1b* alleles for 4 weeks under SD conditions, and then transferred one-third of the plants to LD, one-third to SD, and one-third to night break conditions (NB; 16 h night interrupted at 8 h by 15 min light). Three weeks later, we transferred all plants to continuous light for 24 h, and collected samples every 4 h in the second 24 h period under continuous light (Fig. 5A). *FT2* showed circadian profiles similar to those reported before for *FT1* (Pearce *et al.*, 2017). Transcript levels of *FT2* were very low under SD and high under NB and LD conditions, even in the middle of the night before the NB light pulse (Fig. 5A). However, Kronos lines with no functional *Ppd1* allele (*ppd1*-null; Pearce *et al.*, 2017) showed significantly lower *FT2* transcript levels than those with the functional *Ppd-A1b* allele (Fig. 5B). A similar result was reported before for *FT1* (Pearce *et al.*, 2017), which indicates that *PPD1* acts as a positive transcriptional regulator of both *FT1* and *FT2*.

We also compared the effect of the two functional *PPD1* alleles on *FT1* and *FT2* transcripts under SD conditions. Plants carrying the *Ppd1a* allele (which is expressed under SD conditions at higher levels than *Ppd1b*) showed significantly higher transcript levels of *FT1* relative to *FT2* during the first 8 weeks and similar levels at 10 weeks (Fig. 5C). By contrast, *FT1* transcripts were not detected in the plants carrying the photoperiod-sensitive *Ppd1b* allele during the 10 weeks of the experiment (Fig. 5D). In the same genotype, *FT2* transcripts showed a slight increase from the sixth week and were significantly different from zero by week 10 (Fig. 5D), but they were still more than 10-fold lower than the levels observed in the *Ppd1a* plants at the end of the experiment (Fig. 5C). These results confirmed that *PPD1* acts as a positive regulator of *FT2* transcription and suggest that *FT2* may work as a backup to induce flowering under SD conditions when *FT1* is not expressed.

### Expression of FT2 in the developing spike

**Fig. 6.**
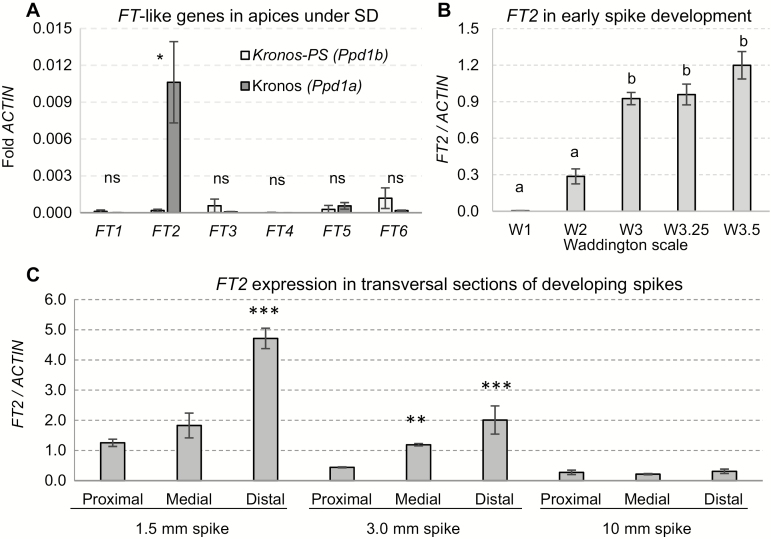
*FT*-like transcript levels in apices and developing spikes. (A) Transcript levels of *FT*-like genes in apical meristems of 8-week-old plants grown under SD. (B) *FT2* transcript levels during early spike development. Waddington scale: W1, vegetative stage; W2, double ridge stage; W3, glume primordium present; W3.25, lemma primordium present; W3.5, floret primordium present. (C) *FT2* transcript levels in transversal sections of the spike at the floret primordia (~1.5 mm), awn elongation (~3 mm), and young spike (~10 mm) stages. Error bars represent standard errors. *P<0.05, **P<0.01, ***P<0.001 (Dunnett’s test against the proximal section in each stage).

We first determined the transcript levels of *FT2* in the SAM from 8-week-old Kronos plants (*Ppd1a*) grown under SD conditions, the time at which we started to detect *FT2* transcripts in the leaves (Fig. 5C). Among the six *FT*-like genes studied, only *FT2* was expressed at levels significantly different from zero (Fig. 6A). As spike development progressed from the double ridge to the floret primordium stage, we detected a rapid increase in *FT2* transcript levels in the developing spike (Fig. 6B).

We then dissected the developing spikes in three sections (proximal, medial, and apical) and measured the expression of *FT2* at the floret primordia (~1.5 mm), awn elongation (~3 mm), and young spike (~10 mm) stages. No significant differences between sections were detected in the latter stage, but in the two early stages, we detected significantly higher levels of *FT2* expression in the distal section of the developing spikes (Fig. 6C).

## Discussion

In this study, we show that *FT2* has a significant effect on spike development and fertility and a more limited role in the transition between the vegetative and reproductive shoot apical meristem.

### Effect of *FT2* on heading time

The overexpression of *FT2* in *B. distachyon* and barley had a dramatic effect on heading time, with spikes emerging almost immediately after callus differentiation. However, it is difficult to interpret these overexpression results, since strong constitutive promoters can drive gene expression in tissues and/or developmental stages that are not observed in natural conditions. We did not obtain seeds from these plants, so we were not able to determine if the rapid flowering was a direct effect of *FT2* or an indirect effect of the up-regulation of other *FT*-like genes.

The milder effects on heading time in barley and *B. distachyon FT2*-RNAi transgenic plants (Fig. 1) and in *ft-A2* mutants in tetraploid wheat reflect better the effect of this gene on heading time in nature. The average delays in heading time varied from 8 d in *B. distachyon* to 4 d in barley and 2–4 d in the wheat *ft2*-null plants. These delays are roughly 10-fold smaller than the ones observed in the wheat *ft1*-mut mutants and the *B. distachyon FT1*-RNAi transgenic plants (Lv *et al.*, 2014). The absence of significant differences in the number of leaves between the plants carrying the *FT2* mutant and wild-type alleles confirmed a limited role of this gene on the timing of the transition of the SAM between the vegetative and reproductive phase. The 2–4 d delay in heading time observed in the wheat *ft2*-null mutants may reflect the additional time required to develop additional spikelets observed in these mutants (Fig. 3) or be an indirect effect of the 46% reduction in *FT1* transcripts (Fig. 4B and Supplementary Fig. S4C).

The relatively smaller effect of the *ft2*-null mutants on heading time is consistent with the up-regulation of *FT2* in the leaves 2–3 weeks later than *FT1* (Fig. 4C, D), a phenomenon also reported in barley. In this species, *FT1* is expressed in the leaves before the transition of the SAM to the reproductive phase and *FT2* a couple of weeks after the initial SAM transition (Faure *et al.*, 2007). These results suggest a limited role of *FT2* in the regulation of the transition of the SAM from the vegetative to the reproductive phase under LD conditions.

### Regulation of *FT2* by other flowering genes

**Fig. 7.**
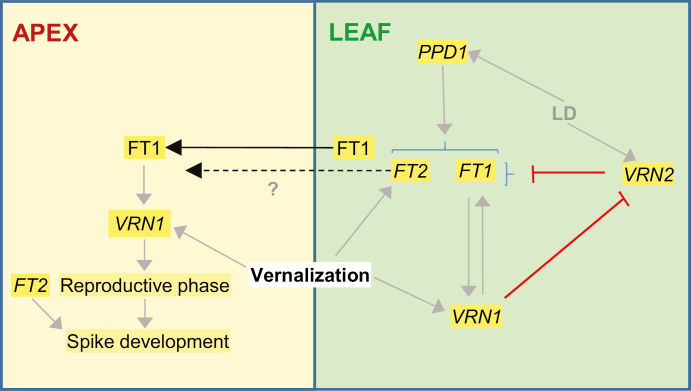
Model describing interactions of *FT1* and *FT2* with other vernalization genes. Both genes are up-regulated by *PPD1* under LD. In winter wheat plants, both genes are repressed under LD by *VRN2* before vernalization. Vernalization results in the up-regulation of *VRN1* and *FT2*, but *FT1* levels remain very low under cold temperatures. The up-regulation of *VRN1* results in the up-regulation of *FT1* both directly and indirectly through the repression of *VRN2.* FT1 then interacts with the floral activation complex, binds to the *VRN1* promoter and further activates *VRN1* transcription generating a positive feedback loop. The up-regulation of *FT1* also has a positive but not immediate effect on the up-regulation of *FT2*. The FT1 protein is then transported to the SAM (we do not know if FT2 is transported or not), where it up-regulates *VRN1* initiating the transition of the SAM to the reproductive stage. *FT2* transcription is up-regulated in the developing spike (mainly in the distal part), where it contributes to the determination of the number of spikelets in the spike. Later, both *FT1* and *FT2* have overlapping roles in the regulation of the number of florets per spikelet and on fertility. Gray arrows indicate promotion and lines with a crossed bar indicate repression. Black arrows indicate transport.

A model for the interactions between *FT2* and other flowering genes is summarized in Fig. 7. The photoperiod experiments demonstrated that *PPD1* is a strong promoter of both *FT1* and *FT2* transcription (Fig. 5). Under short days, both genes showed a stronger up-regulation in the presence of *Ppd1a* than in the presence of *Ppd1b* (Fig. 5C, D), and both genes failed to be up-regulated by night breaks in the *ppd1*-null mutant (Fig. 5B; Pearce *et al.* 2017).

In *B. distachyon*, the microRNA miR5200 is expressed at high levels in the leaves under SD conditions, where it targets *BdFT1* and *BdFT2* mRNAs for cleavage. Disruption of miR5200 activity in this species affects flowering time under SD but not under LD conditions (Wu *et al.*, 2013). We do not know the role of miR5200 in wheat, but it would be interesting to test if the effect of the *PPD1* alleles on *FT1* and *FT2* transcript levels under SD conditions is mediated by miR5200.

The significant reduction in *FT2* transcript levels in the *ft1*-mut mutants (Fig. 4A), together with significant increases in plants overexpressing *FT1* (Lv *et al.* 2014), suggests that *FT1* has a positive effect on the expression of *FT2.* However, this does not seem to be a direct or rapid effect. The increases in *FT1* transcripts observed after *vrn1-null/vrn2*-null plants were returned from vernalization to room temperature were not accompanied by a parallel increase in *FT2* (Fig. 4F). We hypothesize that the frequent correlation observed between *FT1* and *FT2* transcript levels may be an indirect effect of the large changes in plant development induced by *FT1*.

The vernalization experiments (performed under LD conditions) revealed differences in the regulation of *FT1* and *FT2.* In the *vrn1*-null mutants, *FT2* transcript levels increased during vernalization whereas those of *FT1* remained at undetectable levels. Although we cannot rule out a contribution of developmental time to the observed *FT2* increases during vernalization, the down-regulation observed when we returned the plants to room temperature (Fig. 4E, F) suggests a contribution of the low temperature. By the end of the vernalization treatment, *FT2* transcript levels were almost 10-fold higher in the plants carrying the *vrn1*-null and *vrn2*-null mutations than in those carrying only the *vrn1*-null with functional *VRN2* genes. These results suggest that *VRN2* acts as a transcriptional repressor of *FT2* and that vernalization promotes *FT2* transcription independently of *VRN1* and *VRN2* (Fig. 7).

In the leaves of unvernalized *vrn2*-null plants grown under LD conditions, transcript levels of both *FT1* and *FT2* were significantly higher in plants carrying the functional *Vrn-A1* allele than in those carrying the *vrn1*-null alleles (Fig. 4C, D). This result suggests that *VRN1* is able to promote *FT1* and *FT2* transcription independently of *VRN2* (Fig. 7). Direct binding of VRN1 to the *FT1* and *VRN2* promoters has been reported in chromatin immunoprecipitation sequencing experiments in barley (Deng *et al.*, 2015), suggesting a direct regulation of *FT1* and *VRN2* by VRN1 (Fig. 7). We currently do not know if the up-regulation of *FT2* by VRN1 is a direct or indirect effect of the up-regulation of *FT1*.

The up-regulation of *FT2* in the leaves under vernalization or SD conditions, at a time when *FT1* transcript levels are almost undetectable, suggests that *FT2* may serve as a backup to promote wheat flowering under non-inductive conditions. To have an effect on flowering, the encoded FT2 protein would have to travel to the SAM, which has not been demonstrated yet. However, FT2 may not require long distance transport because it is expressed directly in the developing spike, a phenomenon that has not been observed for the other wheat *FT*-like genes studied so far (Fig. 6).

### Effect of FT2 on spike development and fertility

Expression of *FT2* in the SAM was first reported in barley at the time of the floral transition (Digel *et al.*, 2015). Here, we also observed low transcript levels of *FT2* in the SAM in wheat plants grown for 8 weeks under SD conditions (Fig. 6A). *FT2* transcripts increased rapidly during the early stages of spike development under LD conditions. By the time the lemma primordia and the terminal spikelet were formed (Waddington scale 3.5; Waddington *et al.*, 1983), transcript levels of *FT2* were slightly higher than *ACTIN* (Fig. 6B).

Interestingly, transcript levels of *FT2* were highest in the distal part of the early developing spike, where the new lateral spikelets and, eventually, the terminal spikelet are formed in wheat (barley spikes are indeterminate, with no terminal spikelet). The increase in the number of spikelets per spike in the wheat *ft2*-null mutants suggests that this gene plays a role in the regulation of the timing of the formation of the terminal spikelet, which determines the final number of spikelets per spike. Also consistent with this hypothesis is the observation that overexpression of the *OsFTL1* ortholog of this gene in rice results in a drastic reduction in the number of branches in the panicle (Izawa *et al.*, 2002). Overexpression of *FT2* in *B. distachyon* and barley also reduced significantly the number of lateral spikelets (Supplementary Fig. S2), but these results should be considered with caution since we only analysed T_0_ plants.

The *ft1*-mut plants headed significantly later than the *ft2*-null mutations, but the latter showed a stronger effect on the number of spikelets per spike in the same experiments (Supplementary Tables S4–S5). These results, together with their different expression profiles and different biochemical interactions (Li *et al.*, 2015) suggest some degree of sub-functionalization between the wheat *FT1* and *FT2* paralogs. Interestingly, only the mutations in the *FT-A2* homeolog had a significant effect on the number of spikelets per spike, suggesting the existence of some functional differentiation between *FT-A2* and *FT-B2*.

Besides these differences, both *FT1* and *FT2* played similar roles in the regulation of the number of florets per spikelet and floret fertility. Reductions in fertility were observed both in the *FT2*-RNAi transgenic plants in barley and *B. distachyon* (Fig. 1) and in the *ft-A2* and *ft-B2* mutants in wheat (Table 1), which suggests that this *FT2* function may be conserved across the temperate grasses. The significant reductions in fertility associated with the *ft1*-mut and *ft2*-null mutations limit their potential use to increase spikelet number in wheat improvement.

The potential positive effects of the *ft-A2* mutation on grain number (associated with the increases in spikelet and floret number) were overridden by the negative effects caused by its reduced fertility (Table 1). Since *FT1* and *FT2* showed overlapping effects on fertility, the higher transcript levels of *FT1* observed in the *Ft-B1*-Hope allele (Yan *et al.*, 2006; Nitcher *et al.*, 2014) may be useful to compensate the lower fertility of the *ft-A2* mutants. We have previously shown that the *Ft-B1* Hope allele is associated with significant increases in the number of grains per spike relative to the wild-type allele (Nitcher *et al.*, 2014). Another alternative to separate the positive and negative effects associated with *FT2* would be to identify genes that operate downstream of *FT2* and see if they can be modified to increase spikelet number without affecting fertility.

In summary, this study demonstrates that *FT2* plays an important role in spike development and fertility, but a more limited role in the regulation of the SAM transition to the reproductive phase. The expression profile of *FT2* in different photoperiod and vernalization mutants revealed the existence of several interlocked feedback regulatory loops that are likely important to optimize heading time and spike development in different genetic backgrounds and under different environmental conditions. Finally, this study identified positive and negative effects of the *FT2* mutations on different yield components and proposed strategies to separate those effects.

## Supplementary data

Supplementary data are available at *JXB* online.

Fig. S1. Constructs and transformation protocols.

Fig. S2. Overexpression of *FT2* genes in *B. distachyon* and barley.

Fig. S3. Down-regulation of *FT2* transcripts in the RNA interference transgenic *B. distachyon* and barley T_1_ plants.

Fig. S4. Transcript levels of *FT*-like genes and *VRN1* in *FT2*_*RNAi*_ transgenic plants and non-transgenic sibling controls (NTSC) and in *ft2-null* mutants and wild-type control.

Fig. S5. Comparison of the number of florets per spike in wild-type (*Ft-A1/Ft-B1 Ft-A2/Ft-B2*), *ft1-mut* (*ft-A1/ft-B1 Ft-A2/Ft-B2*) and *ft1-mut/ft2-null* (*ft-A1/ft-B1 ft-A2/ft-B2*).

Fig. S6. Alternative splice variants of *FT-A2* and *FT-B2* in wheat leaves.

Table S1. Barley primers for quantitative real-time PCR.

Table S2. *Brachypodium distachyon* heading time and fertility in T_1_*FT2*_*RNAi*_ plants

Table S3. *Hordeum vulgare* heading time, number of florets and grain per spike, and fertility in T_1_*FT2*_*RNAi*_ plants.

Table S4. Effect of *FT1* mutations on heading time and spike parameters (growth chamber under LD).

Table S5. Effect of *FT2* mutations on spike parameters in the presence of the *ft1*-mut mutant (growth chamber under LD).

Table S6. Effect of *FT2* mutations on spike parameters in the presence of the wild-type *Ft1* allele (growth chamber under LD).

Table S7. Effect of *FT2* mutations on spike parameters in the presence of the wild-type *Ft1* allele in the field.

Supplementary Figures S1-S6 and Tables S1-S7
