# Supplementary material for: FLOWERING LOCUS T2 regulates spike development and fertility in temperate cereals
Source: J Exp Bot. 2018 Oct 5;70(1):193–204. doi: 10.1093/jxb/ery350 (PMC6305198; doi:10.1093/jxb/ery350)
Supplement: Supplementary Figures S1-S6 and Tables S1-S7 [file ery350_suppl_supplementary_figures_s1-s6_tables_s1-s7.pdf]

Supplementary data

Supplementary Fig. S1. Constructs and transformation protocols. We cloned the full-length cDNA of *Brachypodium distachyon* Bd21-3 *FT2* (Bradi2g07070) and overexpressed it in the (A) binary vector pCAMBIA1300 under the maize *UBIQUITIN* promoter (*Ubi*) and in the (B) binary vector GWB5 under the 35S promoter and fused to the green fluorescent protein (GFP). (C) We cloned the full-length cDNA of wheat *FT-D2* and overexpressed it in the pGWB17 vector under the maize *UBIQUITIN* promoter and transformed it using *Agrobacterium* into barley cultivar Golden Promise. (D) Two RNAi constructs to silence *FT2* were developed in pCAMBIA1300-based vector driven by the maize *Ubi* promoter. For the RNAi silencing of *FT2*, we selected a 473 bp cDNA fragment (position 47 to 519) from *B. distachyon* and a 216-bp cDNA fragment (position 187 to 402) from barley cultivar Morex. These regions did not share more than 18 identical consecutive nucleotides with other FT-like genes, preventing co-silencing. Tissue culture and *Agrobacterium*-mediated transformation (strain AGL1) were conducted as reported by Bragg *et al.* (2012) and Harwood *et al.* (2009). Black and grey triangles indicate left (LB) and right (RB) borders of the T-DNA, respectively

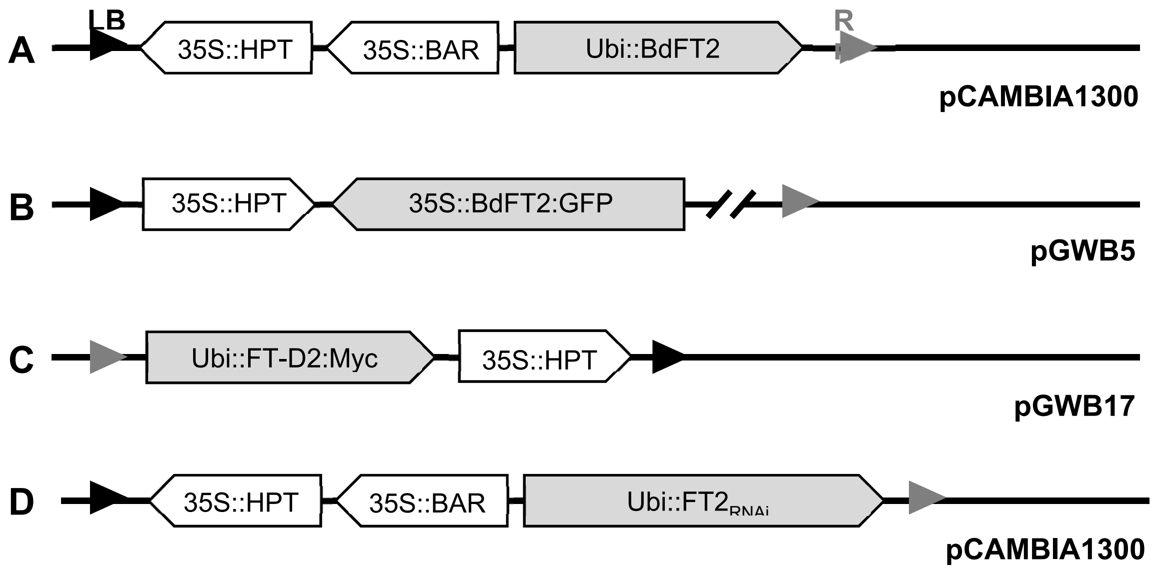

Bragg JN, Wu JJ, Gordon SP, Guttman ME, Thilmony R, Lazo GR, Gu YQ, Vogel JP. 2012. Generation and Characterization of the Western Regional Research Center Brachypodium T-DNA Insertional Mutant Collection. Plos One **7**.

Harwood WA, Bartlett JG, Alves SC, Perry M, Smedley MA, Leyland N, Snape JW. 2009. Barley transformation using *Agrobacterium*-mediated techniques. In: Jones DH, Shewry RP, eds. Transgenic Wheat, Barley And Oats: Production And Characterization Protocols, Vol. 478. Totowa, NJ: Humana Press, 137-147.

Supplementary Fig. S2: Overexpression of the *FT2* gene in *B. distachyon* (A-C) and barley (D-E). (A) Spike formation on regenerated shoots in Ubi::BdFT2 transgenic *B. distachyon* plants. (B) Spikelet formation on regenerated shoots in 35S::BdFT2:GFP transgenic *B. distachyon* plants. (C) Shoot regeneration of the wild type *B. distachyon* control. (D-F) Two independent transgenic events in barley transformed with wheat *FT-D2* (Ubi::FT-D2:Myc) showed a single tiller that flowered very early. Both *B. distachyon* and barley transgenic plants showed spikes with reduced number of spikelets, usually consisting of a single spikelet with multiple florets. All transgenic plants were sterile. Red arrows indicate spikes with a single spikelet. Scale bar =1 cm.

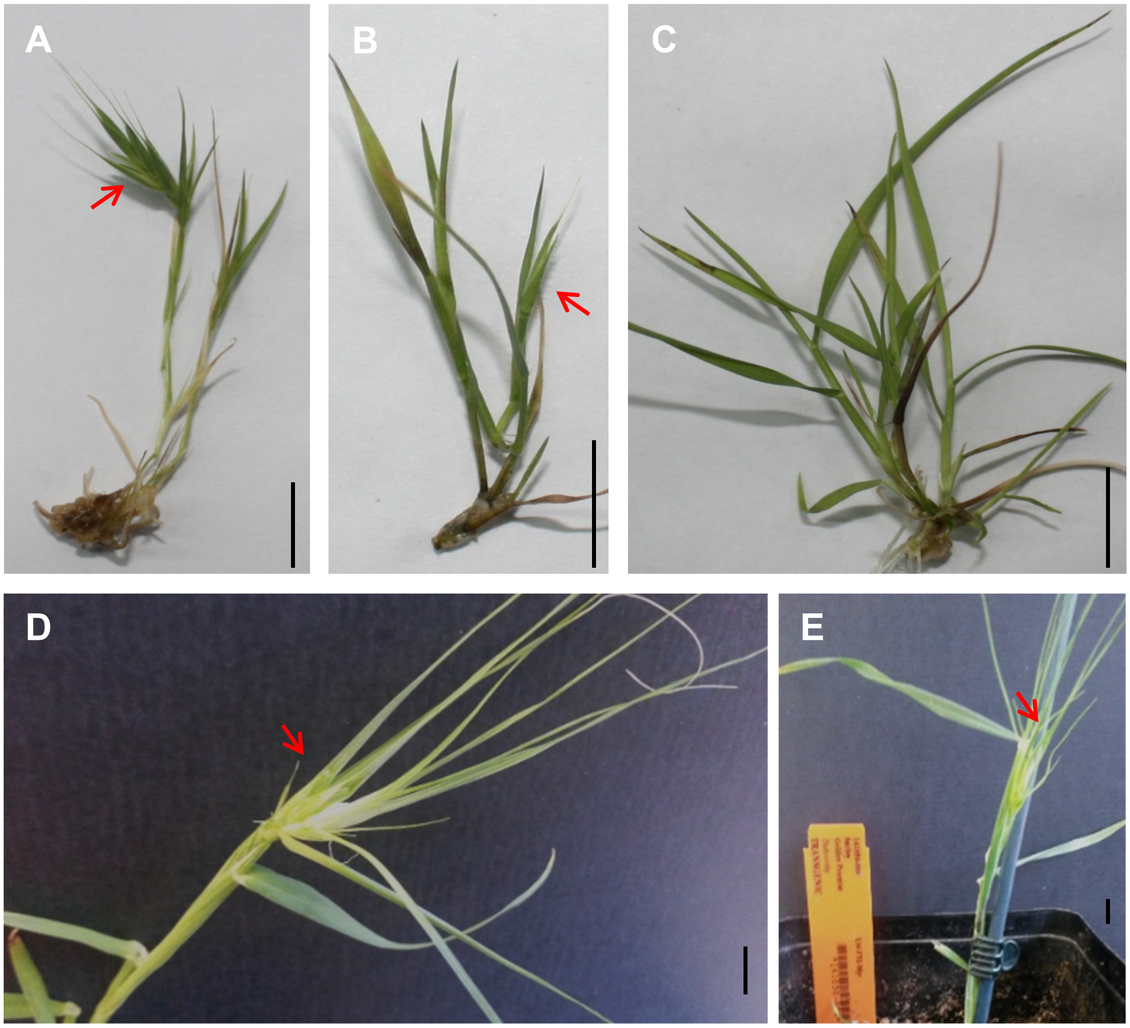

Supplementary Fig. S3: Downregulation of *FT2* transcripts in RNAi transgenic *B. distachyon* and barley T_1 plants. (A) *B. distachyon* T_1 plants with (RNAi) and non-transgenic sibling control (NTSC) in three independent transformation events (700, 693 and 698). *ACTIN* was used as the internal control. (B) Sibling T_1 barley plants with and without the Ubi::$FT2_{RNAi}$ transgene. Leaf samples from both species were harvested five hours after the lights were turned on, one week before wild-type control plants began to flower. Error bars indicate standard errors. Asterisks indicate *P* values: ** = *P* < 0.01, *** = *P* < 0.001.

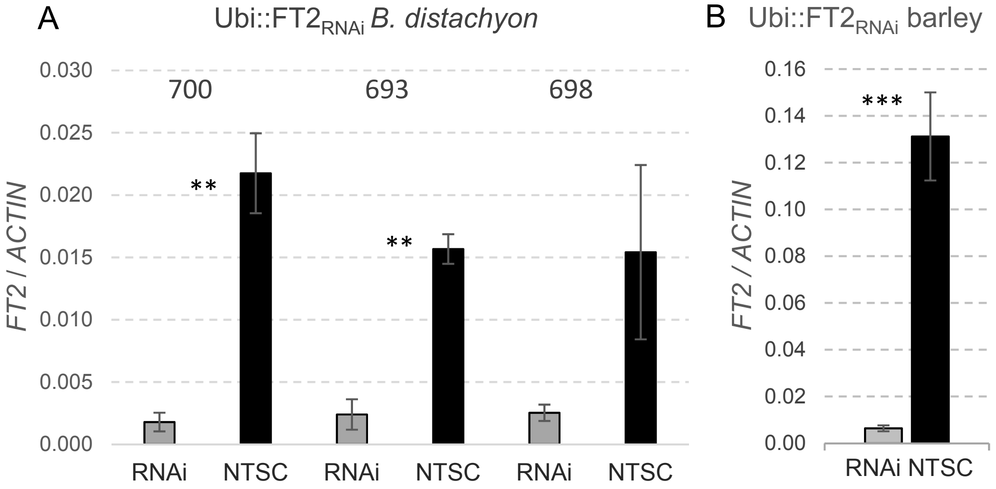

Supplementary Fig. S4: Transcript levels of *FT*-like genes and *VRN1* in *FT2RNAi* transgenic plants and non-transgenic sibling controls (NTSC) (A) *B. distachyon* (n=8). (B) Barley (RNAi n=42, NTSC n=6). (C) Wheat *ft2*-null mutants and sibling wild-type controls (WT) after 5 weeks in LD. Average transcript levels are relative to the *ACTIN* endogenous control calculated using $2^{-\Delta Ct}$. Error bars indicate standard errors. * = $P < 0.05$, ***=P <0.001 (data was transformed when necessary to restore normality and homogeneity of variances). *B. distachyon* and barley leaves were harvested one week before wild-type control plants began to flower. For all experiments, leaves were harvested 3-5 h after lights were turned on.

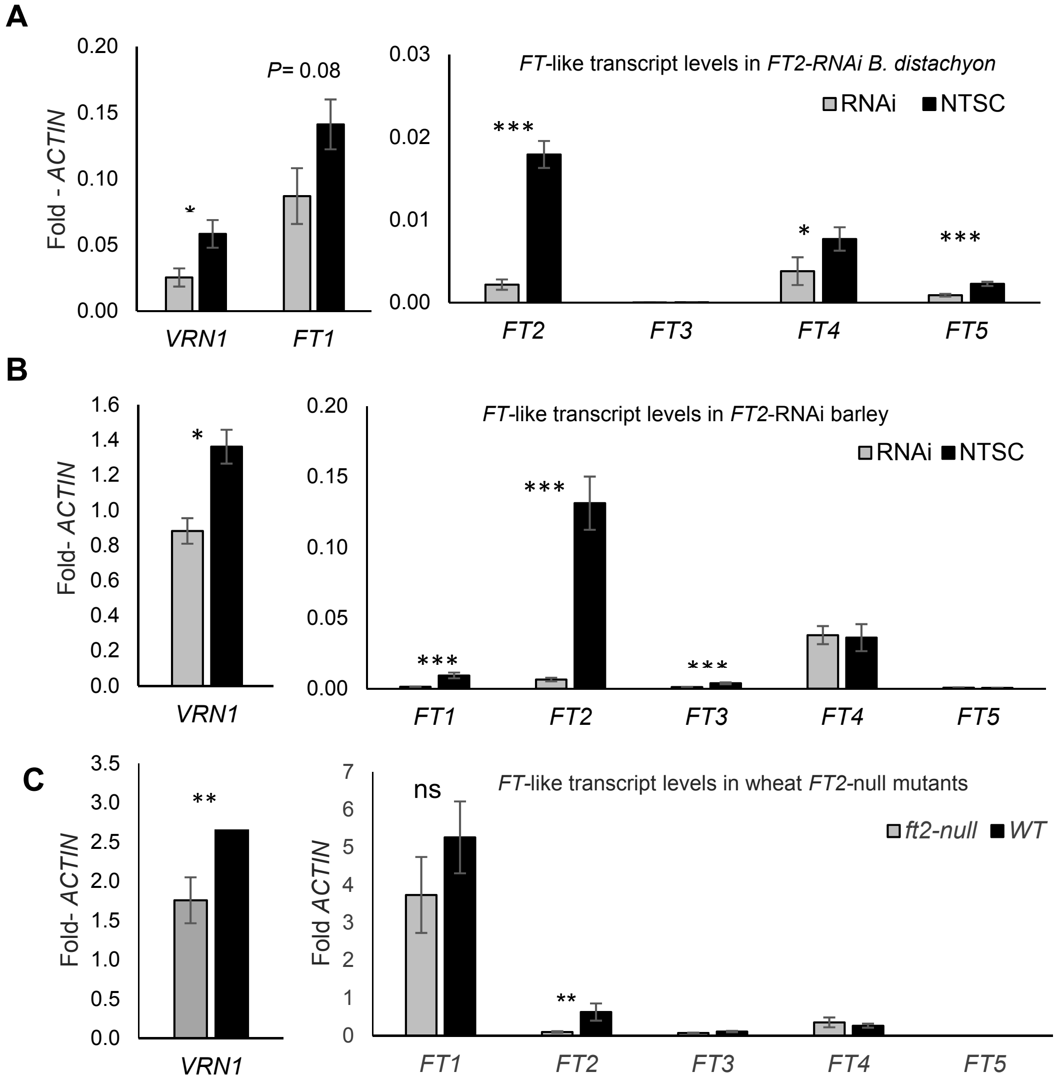

Supplementary Fig. S5: Comparison of the number of florets per spike in wild-type (*Ft-A1/Ft-B1 Ft-A2/Ft-B2*), *ft1*-mut (*ft-A1/ft-B1 Ft-A2/Ft-B2*) and *ft1*-mut/*ft2*-null (*ft-A1/ft-B1 ft-A2/ft-B2*). The *ft1*-mut and the *ft1*-mut/*ft2*-null plants showed 24.5% and 37% more florets per spike than the wild-type, respectively (LD chamber experiment).

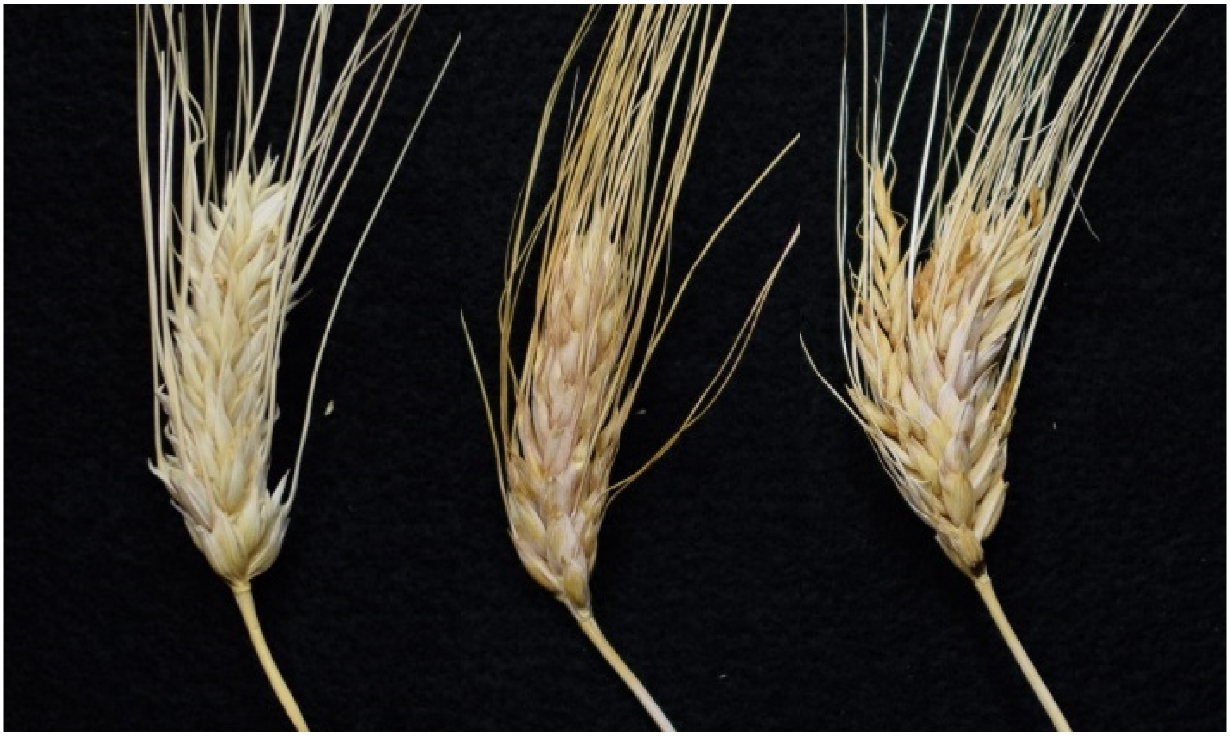

Ft-A1/Ft-B1
Ft-A2/Ft-B2

ft-A1/ft-B1
Ft-A2/Ft-B2

ft-A1/ft-B1
ft-A2/ft-B2

Supplementary Fig. S6: Alternative splice variants of *FT-A2* and *FT-B2* in leaves from three and six weeks-old wheat plants (first two panels). The splice variant encoding the complete protein (blue arrows) was more abundant than the shorter alternative splice variant (red arrows). Lanes between the ladder and the samples have been removed to simplify the image.

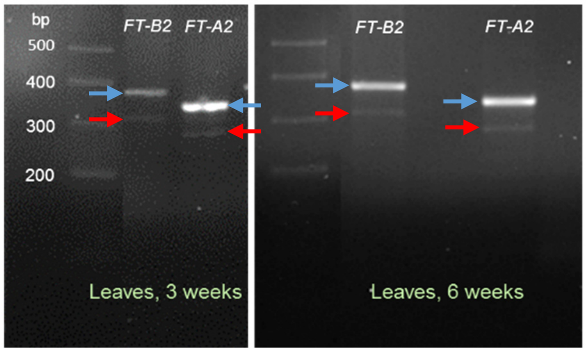

SUPPLEMENTARY TABLES

Supplementary Table S1. Barley primers for quantitative real-time PCR

Gene	Gene bank	Forward Primer (5'to 3')	Reverse Primer (5' to 3')	Primer efficiency
HvFT1	DQ100327	TGTTCTAAGAAGGAAGGAGAAATGG	TCACCCTGAGGTTGGTGGTT	108%
HvFT2	DQ297407	GCGAGCTTCGACAAGACAAAA	GCCGGCGAGCAGGTAGA	100%
HvFT3	DQ411319	TGACAAAATAACAGACAAGGCTAAGG	GGATCCGCTGCAGACATATACTG	107%
HvFT4	DQ411320	CAATGGCATGGAGTTTCGCT	GGATTAGGCGCATCAGGATC	93%
HvFT5	EF012202	TTTTTGCGCCGCACATG	TCTTCCGCCCGATCCA	98%
HvVRN1	AY785825	CTGAAGGCGAAGGTTGAGACA	GCTGCTCCAGTTGCTGCAA	95%
HvGI	AY740523	CGTGCTACAGATGGGATGCTT	AGGAGCTCTAACTGAGGCAAGGT	95%
HvACT [a]	AK362208	TCGCAACTTAGAAGCACTTCCG	AAGTACAGTGTCTGGATTGGAGGG	98%

[a] Yan, L., Fu, D., Li, C., Blechl, A., Tranquilli, G., Bonafede, M., et al. (2006) The wheat and barley vernalization gene VRN3 is an orthologue of FT. Proceedings of the National Academy of Sciences, 103, 19581-19586.

Supplementary Table S2. *Brachypodium distachyon* heading time and fertility in T_1 *FT2*$_{RNAi}$ transgenic and non-transgenic sibling control (NTSC) plants. Transgenic plants were confirmed by resistant to herbicide and/or PCR amplification of the GUS linker region. Averages from the independent events were used as replications in the ANOVA provided in the last row.

Event ID	No. T_1 plants	Genotype	Days to heading	Florets / plant [1]	Grains / plant [1]	Fertility
678	1	Transgenic	83.0	99.0	31.0	31.3%
693	5	Transgenic	78.0	22.5	9.8	38.5%
700	3	Transgenic	79.3	54.9	12.2	22.0%
678	10	NTSC	70.9	14.1	7.3	53.4%
693	5	NTSC	71.4	26.4	13.3	50.8%
700	3	NTSC	71.0	34.9	22.8	65.4%
Average	9	Transgenic	79.0	58.8	17.7	30.6%
	18	NTSC	71.1	25.2	14.5	56.5%
ANOVA			**0.004**	0.217	0.710	**0.017**

[1] We did not registered the number of spikes per plant so we were not able to calculate the number of florets or grains per spike.

Supplementary Table S3. *Hordeum vulgare* (Golden Promise) heading time, number of florets and grains per spike, and fertility in T_1 *$FT2_{RNAi}$* and non-transgenic sibling control (NTSC) plants. Transgenic plants were confirmed by resistant to herbicide and/or PCR amplification of the GUS linker region. Averages from the independent events were used as replications in the ANOVA provided in the last row.

Event ID	No. T_1 plants		Days to heading	Florets /spike	Grains /spike	Fertility
2A	6	Transgenic [1]	106.5	21.1	8.2	43.1%
2B	2	Transgenic	103.5	18.0	7.4	45.2%
1B	5	Transgenic	107.0	20.3	10.0	50.0%
1C	6	Transgenic	106.2	21.1	11.2	52.8%
2-B	3	Transgenic	104.5	21.9	8.7	44.1%
2C	4	Transgenic	103.0	23.2	11.2	47.6%
2D	7	Transgenic	103.1	20.5	7.5	37.2%
2F	6	Transgenic	106.2	21.4	8.6	39.7%
2G	8	Transgenic	108.0	18.3	8.6	46.1%
2H	7	Transgenic	103.0	20.9	11.2	54.7%
2I	7	Transgenic	102.6	18.5	8.9	48.5%
Negative	1	NTSC	101.0	17.8	14.1	79.6%
Negative	1	NTSC	101.0	19.1	10.4	54.2%
Negative	1	NTSC	100.0	21.2	13.9	65.8%
Negative	1	NTSC	102.0	26.5	17.3	65.2%
Average	61	Transgenic	105.0	20.5	9.2	45.6%
	4	NTSC	101.0	21.2	13.9	66.2%
ANOVA			**0.0023**	0.6272	**0.0008**	**0.0003**

[1] Transgenic plants were confirmed by resistant to herbicide and/or PCR amplification of the GUS linker region.

Supplementary Table S4. Effect of *FT1* mutations on heading time and spike parameters in a growth chamber experiment under long day conditions.

LD CHAMBER	HD	SPL	SPS	FLS	GRS	FERT %
**LS means**						
Ft-A1 Ft-B1	44.2	5.34	13.63	4.25	31.0	51.8 %
ft-A1 ft-B1	65.4	5.85	13.33	5.29	17.2	24.1 %
**ANOVA for *FT1***						
Ft1 vs. *ft1*-mut	**<0.0001**	0.3070	0.7352	**0.0228**	**0.0319**	**0.0003**

*HD: days to heading, SPL: spikelet length in cm, SPS: number of spikelets per spike, FLS: number of florets per spike, GRS: number of grains per spike, FERT: percentage fertility.

Supplementary Table S5. Effect of *FT2* mutations on spike parameters in plants with an *ft1*-mut mutant background. Growth chamber under LD.

LD CHAMBER	*FT1*	SPL	SPS	FLS	GRS	FERT %
**LS means**						
Ft-A2 Ft-B2	*ft1*-mut	5.85	13.33	5.29	17.2	24.1 %
ft-A2 Ft-B2	*ft1*-mut	6.60	15.38	6.00	30.4	33.9 %
Ft-A2 ft-B2	*ft1*-mut	6.25	14.67	5.58	19.8	23.8 %
ft-A2 *ft-B2*	*ft1*-mut	6.13	15.50	7.25	6.7	6.5 %
**Effects in *ft1*-mut**						
ft-A2/Ft-A2	*ft1*-mut	5.2%	**10.3%**	**21.8%**	0.1%	-15.7%
ft-B2/Ft-B2	*ft1*-mut	-0.5%	5.1%	**13.7%**	**-44.3%**	**-47.8%**
ftA2ftB2/FtA2FtB2	*ft1*-mut	4.8%	16.3%	37.0%	**-61.2%**	**-73.0%**
**ANOVA for *FT2* in *ft1*-mut**						
FT-A2 P	*ft1*-mut	0.1667	**0.0356**	**<0.0001**	0.9961	0.4424
FT-B2 P	*ft1*-mut	0.8817	0.2683	**0.0029**	**0.0212**	**0.0087**
Interaction	*ft1*-mut	0.0632	0.3569	0.0744	**0.0051**	**0.0098**

* SPL: spikelet length in cm, SPS: Number of spikelets per spike, FLS: Number of florets per spike, GRS: number of grains per spike, FERT: fertility in percent.

Supplementary Table S6. Effect of *FT2* mutations on spike parameters in plants with a wild-type *Ft1* allele background. Growth chamber under LD.

LD CHAMBER	SPL*	SPS	FLS	GRS	GW	FERT%
**LS means**						
Ft-A2 Ft-B2	5.071	13.071	4.100	16.914	57.557	31.257
ft-A2 Ft-B2	5.383	16.167	4.467	23.500	58.317	32.683
Ft-A2 ft-B2	5.258	14.492	4.050	18.892	58.808	30.775
ft-A2 *ft-B2*	5.033	15.608	4.333	15.892	52.117	23.825
**Effects of *FT2***						
ft-A2/Ft-A2	0.8%	**15.3%**	**8.0%**	10.0%	-5.1 %	-8.9%
ft-B2/Ft-B2	-1.6%	2.9%	-2.1%	-32.5%	-4.3 %	-14.6%
ftA2ftB2/FtA2FtB2	-0.7%	19.4%	5.7%	**-6.0%**	-9.5 %	-23.8%
**ANOVA *FT-A2 x FT-B2***						
FT-A2 P	0.8149	**0.0008**	**0.0346**	0.4022	0.4531	0.3890
FT-B2 P	0.7130	0.4529	0.5385	0.1918	0.3566	0.1494
Interaction	0.2212	0.0905	0.7793	**0.0300**	0.2820	0.1947

* SPL: spikelet length in cm, SPS: Number of spikelets per spike, FLS: Number of florets per spike, GRS: number of grains per spike, GW= grain weight in mg, FERT: fertility in percent.

Supplementary Table S7. Effect of *FT2* mutations on spike parameters in plants with a wild-type *Ft1* allele background. Experiment performed in the field.

LD CHAMBER	SPL*	SPS	FLS	GRS	GW	FERT%
**LS means**						
Ft-A2 Ft-B2	8.163	20.688	4.875	63.313	61.505	62.991
ft-A2 Ft-B2	7.769	21.688	4.859	54.167	54.863	51.584
Ft-A2 ft-B2	7.927	19.667	5.104	50.302	57.382	50.077
ft-A2 *ft-B2*	8.315	23.646	5.344	53.479	56.634	42.631
**Effects**						
ft-A2/Ft-A2	0.0%	**12.3%**	2.2%	-5.3%	**-6.2%**	**-16.7%**
ft-B2/Ft-B2	1.9%	2.2%	**7.3%**	**-11.7%**	-2.0%	**-19.1%**
ftA2ftB2/FtA2FtB2	1.9%	**14.3%**	9.6%	**-15.5%**	**-7.9%**	-32.3%
**ANOVA**						
FT-A2 P	0.9771	**<0.0001**	0.3001	0.3639	**0.0048**	**0.0010**
FT-B2 P	0.1617	0.0940	**0.0025**	**0.0336**	0.3325	**0.0002**
Interaction	0.0013	**<0.0001**	0.2392	**0.0470**	**0.0206**	0.4401

* SPL: spikelet length in cm, SPS: Number of spikelets per spike, FLS: Number of florets per spike, GRS: number of grains per spike, FERT: fertility in percent.
